# Evaluation of nutritional and phytochemical variability of cowpea Recombinant Inbred Lines under contrasting soil moisture conditions in the Guinea and Sudan Savanna Agro-ecologies

**DOI:** 10.1016/j.heliyon.2020.e03406

**Published:** 2020-02-18

**Authors:** M.S. Alidu, I.K. Asante, H.K. Mensah

**Affiliations:** a Department of Agronomy, Faculty of Agriculture, University for Development Studies, P. O. Box TL, 1882, Nyankpala, Tamale, Ghana; b Department of Plant and Environmental Biology, University of Ghana, P.O. Box LG 57 Legon, Accra, Ghana

**Keywords:** Agriculture, Plant biology, Polyphenols, Cowpea, Biochemical analysis, Correlation and regression analysis

## Abstract

Plant breeders’ efforts in developing drought tolerant and high-yielding cowpea varieties may be in vain unless the developed varieties are evaluated to ascertain the influence of water stress on their nutritive value, antioxidants, and phenolic contents under contrasting moisture regimes. The study was set up to evaluate the nutritional value, phytochemical content and antioxidant activity of cowpea Recombinant Inbred Lines (RILs) under contrasting soil moisture in the Guinea and Sudan Savanna agro-ecologies of Ghana. Forty-eight cowpea RILs seed samples from well-watered and water stress experiment were pulverized. Distillation and titration was carried out and the organic samples extracted and various biochemical analyses were carried out using standard protocols and methodologies. The dried grain mineral contents were determined using an Atomic Absorption Spectrophotometer, while the protein content was determined by the combustion method. Correlation and regression analysis and principal component analysis were performed using STATA version 13. Biochemical analysis for seed related traits revealed that inbred lines responded differently to drought. Significant differences of watering regimes on various phytochemical traits were only observed in phosphorus and lead. Inbred line with family number **57** had the highest crude protein content of 46.90% under well-watered conditions. Inbred line **84** under water stress conditions had high levels of Mg and K. Inbred line **20** under well-watered conditions had the highest antioxidant content. For phenolic acid content, inbred line **255** scored the highest. Quercetin and rutin were most abundant in inbred line **186** for both water-stress and well-watered conditions. The indication is that moisture stress could affect seed yield but no effects on the phytochemical and nutritional variables. Genotypic differences could arise from parental combination used for developing the inbred lines for the study.

## 1 Introduction

Cowpea [*Vigna unguiculate* (L.) Walp.] is an extensively cultivated legume food crop of the tropics and sub-tropics, used in the diets of humans and animals. Cowpea seeds are an excellent source of carbohydrate (50–60%) and an essential source of protein (18–35%) (Stancheva et al., 2017; Addo-Quaye et al., 2011). Cowpea also contains considerable amount of micronutrients such as vitamin A, iron, Zinc and calcium (Quaye et al., 2009; Prinyawiwatkul et al., 1996). The crude protein from the seeds and leaves ranges from 23 to 32% (Diouf, 2011). Since cowpea is an essential source of protein for all, the effects of moisture stress on protein and free amino acid concentrations in seeds cannot be over emphasized. Also, the leaves and stems have been noted for its high amounts of Ca, Mg, K and Zn and therefore may serve as an integral mineral source for both animals and soil amendments to improve the fertility for enhanced crop productivity (Hu, 1981).

Cowpea can also be used to supplement other meals for children to provide them with adequate mineral requirements. Cowpea being an important grain and multi-purpose legume is a source of food and protein for both human and livestock. Cowpea is fed to animals in different feed formulations and the forage form, can have crude protein of about 22% and therefore is highly commended as a supplementary protein feed for animals on low quality diets (Gwanzura et al., 2012; Paduano et al., 1995). Reports from various study on cowpea indicates that the haulms could be used to sustain animal growth and milk production in lactating dairy cattle during the dry season (Anele et al., 2010, 2011). Plants in their natural environment are exposed to several abiotic stresses, affecting its growth and productivity (Osman and El-Gawad, 2013). Farooq et al. (2013), reported that drought is the most devastating environmental stress, which decreases crop productivity, affecting seed yield more than any other environmental stress. Nutrients require water for uptake and translocation. Therefore as water supply declines, nutrient uptake is supressed, and consequently these will affect the rate of cell division and elongation, leaf area, root and stem growth, interrupted stomatal conductance, and water use efficiency (Farooq et al., 2009), and that may arise when cowpea is grown under water stress conditions. Also, Lecoeur and Guilioni (1998) reported that, severe soil moisture deficit results in reduction in protein content as well as modifications in composition of nutrients. The chemical composition and nutritional properties of cowpeas vary considerably according to cultivar (Giami, 2005; Rangel et al., 2004). For effective utilization of newly developed cowpea cultivars for human and/or animal nutrition, evaluation of their nutritional properties are necessary (Giami, 2005). However, little attention has been paid to the possible variations of the essential nutrients such as protein and amino acids among others (Akinyele and Abudu, 1990). Efforts by Breeders will be in vain if improved varietal development programs do not include investigations into the nutritional components of the newly developed varieties for food security (Punia and Darshan, 2000).

The study was designed to evaluate the nutritional value, phytochemical content and antioxidant activity of cowpea inbred lines under contrasting soil moisture in the Guinea and Sudan Savanna agro-ecologies in Ghana.

## 2 Materials and methods

### 2.1 Source of cowpea accessions

Four hundred and fifty (450) Recombinant Inbred Lines (RILs) of cowpea seeds were developed from a cross between drought tolerant and susceptible parents. They were advanced through single plant selection until an F_6_ generation was reached. This population was screened for seedling tolerance to drought in Screen-house conditions over a two-phase period. Thereafter, potential seedling tolerant and susceptible inbred lines were selected and subsequently evaluated under managed stress conditions for two growing drought conditions under Irrigation facilities in Tamale in the Northern Guinea and Sudan Savanna ecologies. The parents were obtained from IITA, Kano, Nigeria. 24 RILs of cowpea seed samples were each taken from stress and non-stress experiments respectively.

### 2.2 Preparation of cowpea flour

One hundred (100) cowpea seeds from each inbred line were pulverized using an electronic miller. The flour was then stored in plastic bags and kept in airtight cupboards until required for use.

### 2.3 Determination of protein content

The sample digestion was done using the Kjedahl method described in AOAC (2000).

The determination of nitrogen was done using the Kjeldahl method described in AOAC (2000). The Kjedahl method involves two major procedures: distillation and titration. Five millilitres (5ml) of the digested samples for each accession was measured and transferred into 50ml conical flasks. Five millilitres (5 ml) of 2% boric acid solution was added, thus changing the colour to purple. The digestion tube was then fixed to the distilling end in the presence of 5ml of 40% NaOH solution. The colour of the sample was thus changed from purple to light green. The distillate was then titred against 0.1 M HCl until a colour change was noted. The titre values were recorded for each accession. The entire procedure was repeated using ammonia free distilled water as blank.

Calculation of percentage nitrogen was done using the formula:

$$\%N=\frac{titre\;value\times 0.01\times 14\times V\times 100}{1000\times W\times aliquot\;pipetted}$$

(AOAC, 2000).where *N* represents nitrogen, *V* represents the extraction volume (100ml) and *W* represents the weight of the powdered sample (0.1g).

Percent crude protein was calculated by using the formula:

% Crude Protein = %N x 6.25 (AOAC, 2000).

### 2.4 Quantification of phosphorus

The procedure used was the Ascorbic acid method for testing phosphorus(Siddhuraju and Becker, 2007). For each of the samples, clean 50 ml volumetric flasks were filled with distilled water to the 25-ml mark. One millilitre (1.0ml) of digested sample from each accession was pipetted into the volumetric flasks. A drop of P-Nitrophenol was added to each sample. Drops of ammonia were then added to the sample, until there was colour change (colourless to yellow). A mass of 0.264 g of ascorbic acid was weighed on the electronic balance and added to 50 ml of Reagent A. The solution was stirred with a stirring rod. Five millimetres of the ascorbic acid and Reagent A solution was measured with a measuring cylinder and then added to each sample. The sample changed colour from yellow to colourless, and then finally changed to blue. Distilled water was added up to the 50ml mark of the volumetric flasks. Samples were then shaken gently. Small volumes of samples were discharged into cuvettes and their absorbance were read using the spectrophotometer.

#### 2.4.1 Quantification of K, Ca, Mg, Na, Mn, Ni, Zn and Fe

The following mineral elements: K, Ca, Mg, Na, Mn, Ni, Zn and Fe were detected by using the Atomic Absorption Spectrophotometer (AAS)as in (Karpiuk et al., 2016). The Atomic absorption (AA) spectrometer is used to analyze metals at very low concentrations, typically in the parts per million (ppm) or parts per billion (ppb) ranges. A liquid sample containing dissolved material whose concentration is to be measured is aspirated into a thin, wide AA flame, or is introduced into a small carbon furnace which is heated to a high temperature. The principle of AAS is the measurement of absorption of radiation by free atoms. The total amount of absorption depends on the number of free atoms present and the degree to which the free atoms absorb the radiation. At the high temperature of the AA flame, the sample is broken down into atoms and it is the concentration of these atoms that is measured.

### 2.5 Total phenolic compound analysis

Methanolic extracts from the cowpea flour for each accession were analysed for phenolic compounds using the Folin-Ciocalteu method (Singleton and Rossi, 1965).

Nine polyphenolic compounds were analysed among the 48 cowpea accessions. These polyphenolic compounds are sub grouped into phenols and flavonoids. The phenolic acids were; 2, 5-dihroxybenzoic acid, caffeic acid, chlorogenic acid, gallic acid, p-coumeric acid, syringic acid, vanilic acid; and flavonoids were: rutin and quercetin.

#### 2.5.1 Preparation of sodium carbonate solution

A 50ml volumetric flask was filled with 20ml distilled water. A mass of 6.25g of sodium carbonate was weighed and dissolved in the distilled water. The solution was boiled, allowed to cool and then few crystals of sodium carbonate were added. The solution was made to stand for 24 h and then filtered. Distilled water was added up to the 25ml mark.

#### 2.5.2 Extraction of samples for phytochemical studies

A mass of 0.5g of all the cowpea flour for all accessions was measured using the electronic balance and poured in McCartney bottles. Twenty millilitres (20ml) of 100% methanol was added and shaken. The bottles were covered and allowed to stand for 24 h. After the 24-hour period, the sample in solution was filtered and the filtrate stored in tightly covered McCartney bottles at temperature of 4 °C in the fridge.

#### 2.5.3 Determination of phenolic acid content

Extract of the cowpea flour for each accession were analysed for phenolic compounds using the Folin-Ciocalteu method (Singleton and Rossi, 1965). After the 24-hour period, 1 ml of each extract was measured with a measuring cylinder and then diluted to 10 ml with distilled water in test tubes. Twenty microliters (20μl) of diluted samples were pipetted into cuvettes. A volume of 1.58 ml of distilled water and 100μl Folin-Ciocalteu reagent were measured with a measuring cylinder and a 100μl micropipette and added to the solution. The solution was shaken to mix. A volume of 300μl of sodium carbonate was pipetted and added to the solution after 5 min and shaken. The solution was placed in the oven for 30 min at a temperature of 40 °C. The cuvettes were taken out after the 30 min and allowed to stand for 90 min. The absorbance at 765 nm was determined against the blank methanol using the visible spectrophotometer. The concentration for the phenolic compounds for each accession was determined from the standard curves of linear equations.

#### 2.5.4 Determination of flavonoid content

The modified aluminium chloride colorimetric procedure was used for the determination of flavonoid content in the cowpea samples. A volume of 100μl of samples extract were pipetted and added to 500μl of distilled water and 30 μl of 5% sodium nitrite in cuvettes. The resulting solutions were made to stand for 5 min after which 30μl of aluminium chloride was added. The solutions were allowed to stand again for 6 min after which 200μl of sodium hydroxide and 110μl of distilled water were added to the solutions and vortexed. Measurement of absorbance of the solution for each accession was made at a wavelength of 425 nm for rutin and 415 nm for quercetin using the spectrophotometer. The concentration for individual flavonoid compound was calculated according to their respective standard curves and the results expressed as mg/l of extract.

#### 2.5.5 Standard curves

The standard curves used for the polyphenolic compounds are presented in Table 1.

**Table 1:** Polyphenolic compounds and their standard curves.

Polyphenolic compound	Standard curve
2, 5-dihydroxybenzoic acid	y = 0.0027x – 0.109
caffeic acid	y = 0.0023x -0.1291
chlorogenic acid	y = 0.0019x – 0.076
gallic acid	y = 0.0012x – 0.0065
syringic acid	y = 0.0019x – 0.0722
p-coumeric acid	y = 0.0014x + 0.0334
vanilic acid	y = 0.0019x- 0.0026
Rutin	y = 0.007x + 0.062
Quercetin	y = 0.0008x + 0.1099

#### 2.5.6 Determination of antioxidant activities

An amount of 5μl of DPPH solution was added to 100μl of methanolic sample extracts. An amount of 1 ml of methanolic extract was added to 0.002% DPPH solution. The same amount of sample extract was added to the standard solution to be tested separately. The resulting mixtures were allowed to stand in the dark for 20 min after which optical density was measured at 517nm using spectrophotometer against methanol. Percentage inhibition was calculated from the optical density according to the formula given below:

$$\text{Percentage inhibition of DPPH activity }=\;\frac{\left(\text{A }–\text{ B}\right)}{A}\;\times \;100$$

(Rahman et al., 2012).where A = optical density of the blank and B = optical density of sample.

### 2.6 Data analysis

The phytochemical traits were recorded in the 48 cowpea inbred lines grouped under non-stress and stress conditions. Descriptive statistics, test of means (t-test), pairwise correlation analysis, Regression analysis and principal component analysis were performed using STATA version 13. The descriptive statistics generated were used to provide the means, standard error for the variables. T-test was performed for both conditions and inbred lines used to generate means of phytochemical traits to show significant differences among groups. Pairwise correlation analysis and regression analysis were carried out to determine the significance of association between the variables. Principal component analysis (PCA) was employed to determine the percentage contribution of each trait to total genetic variation.

## 3 Results

### 3.1 Mean crude protein and minerals

The results for means with respect to crude protein and minerals concentration for cowpea inbred lines are presented in Table 2.

**Table 2:** Means for crude protein and mineral concentration of cowpea inbred lines under water-stress and non-stress conditions.

Family	Phosphorus		Crude protein		Magnesium		Potassium		Chromium		Zinc		Iron		Lead		Copper		Manganese		Selenium	
	NS	S	NS	S	NS	S	NS	S	NS	S	NS	S	NS	S	NS	S	NS	S	NS	S	NS	S
84	0.68	1.03	20.84	28.53	7.88	8.37	1.49	1.99	0.03	0.01	0.01	0.01	0.11	0.09	0	0	0.02	0.01	0	0	0.5	0.43
55	1.05	0.9	22.75	19.25	8	7.73	1.49	1.47	0.01	0	0.01	0.01	0.08	0.09	0.14	0	0.02	0	0	0	0.68	0.42
230	0.75	0.85	22.93	18.2	7.9	7.83	1.46	1.21	0.02	0.04	0.01	0.01	0.09	0.17	0.1	0	0.01	0.01	0	0	0.48	0.54
20	0.61	0.46	22.23	16.98	7.63	7.82	1.43	1.47	0	0.01	0.01	0.01	0.05	0.17	0	0.01	0.01	0	0	0	0.57	0.81
255	0.53	0.84	15.75	19.81	7.86	8.27	1.28	1.68	0.01	0.01	0.01	0.01	0.05	0.15	0.02	0	0.01	0	0	0	0.37	0.53
398	0.92	0.73	17.33	21.56	8	8.2	1.63	1.66	0.04	0.02	0.01	0.01	0.09	0.13	0	0.02	0.01	0	0	0	0.36	0.48
279	0.35	1.24	17.17	19.78	7.76	7.87	1.52	1.29	0.01	0.03	0.01	0.01	0.08	0.1	0	0.03	0.01	0.02	0	0	0.6	0.76
28	0.66	0.86	24.33	23.1	7.68	7.45	1.47	1.36	0.02	0.05	0.02	0.01	0.08	0.08	0	0	0.01	0	0	0	0.39	0.25
189	0.77	0.66	16.48	18.38	7.96	7.65	1.47	1.28	0.03	0.03	0.01	0.01	0.04	0.08	0.11	0.01	0.01	0	0	0	0.17	0.58
503	0.32	0.89	20.68	22.93	7.84	8.05	1.31	1.54	0	0.01	0.01	0.01	0.06	0.07	0.08	0.01	0.01	0	0	0	0.4	0.53
116	0.43	1.09	16.45	17.33	7.99	8.06	1.61	1.64	0.04	0.03	0.01	0.01	0.16	0.05	0.11	0.04	0.01	0	0	0	0.28	0.69
142	0.51	0.99	15.42	19.78	7.58	7.89	1.01	1.38	0.02	0.04	0.01	0.01	0	0.13	0.02	0	0	0.01	0	0	0.78	0.59
406	0.93	0.73	21.89	12.6	7.59	7.97	1.21	1.36	0.01	0.04	0.01	0.01	0.04	0.12	0.12	0	0	0.01	0	0	0.43	0.16
78	0.49	0.99	20.13	14.03	7.76	8.13	1.38	1.46	0.03	0.03	0.01	0.01	0.01	0.12	0.05	0	0	0.01	0	0	0.12	0.22
57	0.69	0.76	46.9	23.12	7.9	7.43	1.34	1.1	0.02	0.01	0.01	0.01	0.13	0.11	0	0.02	0	0	0	0	0.06	0.65
353	0.64	0.35	19.43	23.45	8.07	7.97	1.78	1.45	0.04	0.03	0.01	0.01	0.04	0.1	0	0.02	0	0.01	0	0	0.44	0.52
75	0.69	0.23	19.6	16.28	8.1	7.43	1.62	0.99	0.01	0.01	0.01	0.01	0.13	0.08	0	0.03	0	0.01	0	0	0.17	0.35
223	0.58	0.91	21.88	28.89	8.01	8.14	1.56	1.67	0.06	0.04	0.01	0.01	0.1	0.08	0.11	0	0	0	0	0	0.43	0.54
325	0.45	0.29	20.33	23.1	7.48	8.05	1.4	1.4	0.01	0.04	0.01	0.01	0.12	0.07	0	0.05	0	0	0	0.01	0.37	0.51
131	0.32	1.16	16.63	20.86	7.86	7.74	1.45	1.41	0.04	0.02	0.01	0.01	0.12	0.07	0.04	0.08	0	0	0	0	0.23	0.28
38	0.23	1.05	15.58	15.42	7.8	7.66	1.42	1.29	0.03	0	0.01	0.01	0.06	0.07	0.13	0.03	0	0	0	0	0.14	0.39
408	0.38	0.64	19.78	20.48	7.87	7.67	1.48	1.3	0.01	0.04	0.01	0.01	0.06	0.06	0.05	0	0	0	0	0	0.49	0.2
186	0.33	0.34	23.1	23.1	7.9	7.77	1.89	1.32	0.03	0.01	0.01	0.01	0.03	0.05	0.08	0	0	0.01	0	0	0.6	0.39
396	0.66	0.74	18.9	21.18	7.46	7.81	1.19	1.59	0.02	0.01	0.01	0.01	0.07	0.02	0	0.08	0	0.01	0	0	0.58	0.14
Mean	0.58	0.78	20.7	20.3	7.83	7.87	1.46	1.43	0.02	0.02	0.01	0.01	0.08	0.09	0.05	0.02	0.01	0.01	0	0	0.39	0.46
Std. err	0.04	0.06	1.25	0.81	0.37	0.5	0.04	0.04	0	0	0	0	0.01	0.01	0.01	0	0	0	0	0.01	0.04	0.04
CV%	36.1	35.1	30.5	20.8	6.12	7.1	15	16.5	59.4	60.1	34.8	31	50	40	100	100	86.1	**75**	**.**	100	47.2	40.4
Min	0.23	0.23	15.4	12.6	7.46	7.43	1.01	0.99	0	0	0	0	0	0.02	0	0	0	0	0	0	0.06	0.14
Max	1.05	1.24	46..9	28.9	8.1	8.37	1.89	1.99	0.06	0.05	0.02	0.02	0.16	0.17	0.14	0.08	0.02	0.0**2**	0	0.01	0.78	0.81
P > t	**		**ns**		**ns**		**ns**		**ns**		**ns**		**ns**		**		**ns**		**ns**		**ns**	

NS = Non-stress condition S = Stress condition **Statistical significance between groups at P < 0.01 using t‟ test ns = not significant Min = Minimum Max = Maximum.

CV = Coefficient of variation.

### 3.2 Mean crude protein concentration

Mean percent crude protein for non-stress cowpea inbred lines ranged from 15.42 – 46.9% with a grand mean of 20.69%, higher than cowpea inbred lines under stress conditions, which ranged from 12.6 to 28.89% with a grand mean of 20.34%. Comparatively, mean crude protein concentrations between stress and non-stress showed both treatments were not significantly different.

### 3.3 Mean phosphorus concentration

Mean percent phosphorus concentration for non-stress cowpea inbred lines was between 0.23 -1.05% with a grand mean of 0.58%, whereas for cowpea inbred lines under stress conditions concentrations ranged from 0.23 - 1.24% with a grand mean of 0.78%. However, there were significant differences (P < 0.01) between non-stress and stress cowpea inbred lines.

### 3.4 Mean magnesium concentration

Mean percent magnesium concentration for non-stress cowpea inbred lines ranged from 7.46 to 8.1% with a grand mean of 7.83%, while for cowpea inbred lines under stress conditions the values ranged from 7.43-8.37% with a grand mean of 7.87%. Furthermore, mean magnesium concentration between stress and non-stress showed both treatments were not significant.

### 3.5 Mean potassium concentration

Mean percent potassium for non-stress cowpea inbred lines was 1.01%–1.98% with a grand mean of 1.46. Mean potassium concentration for cowpea inbred lines under stress conditions ranged from 0.99% to 1.9% with a grand mean of 1.43%. The reported mean potassium concentrations for both treatments showed that both means were not significantly different.

### 3.6 Mean chromium concentration

The minimum and maximum chromium concentration were 0.00% and 0.06% respectively for cowpea inbred lines under non-stress conditions. However, the mean percent chromium for cowpea inbred lines under stress ranged from 0.000 to 0.05% with a grand mean of 0.02% for both stress and non-stress conditions. However, there were no significant differences between mean chromium concentration under non-stress and stress conditions.

### 3.7 Mean zinc concentration

The mean percent zinc for cowpea inbred lines under both stress and non-stress ranged from of 0.00–0.02% with a grand mean of about 0.01%.

### 3.8 Mean iron concentration

Mean percent iron for non-stress cowpea inbred lines scored between 0.00 - 0.16% with a grand mean of 0.08%, whereas cowpea inbred lines under stress conditions ranged from 0.02 - 0.17% with a grand mean of 0.09%. Mean iron concentrations between stress and non-stress showed that both treatments were not significantly different.

### 3.9 Mean lead concentration

Mean lead concentration for non-stress cowpea inbred lines ranged from 0.00 - 0.14% with a grand mean of 0.05% significantly higher than cowpea inbred lines under stress conditions, which ranged from 0.00 - 0.08% with a grand mean of 0.02%.

### 3.10 Mean copper concentration

Mean percent copper concentration for non-stress cowpea inbred lines ranged from 0.00-0.02% with a grand mean of 0.01%, whereas cowpea inbred lines under stress conditions had values ranging from 0.00-0.02% with a grand mean of 0.01%. The mean copper concentrations between stress and non-stress were not significantly different.

### 3.11 Mean manganese concentration

The minimum and maximum mean percent of manganese was 0.00% for cowpea inbred lines under non-stress condition. Equally, stress and non-stress scored a grand mean value of 0.00% of manganese concentration.

### 3.12 Mean percent selenium concentration

Mean percent selenium concentration for non-stress cowpea inbred lines scored between 0.06 - 0.78% about a grand mean of 0.39%, whereas cowpea inbred lines under stress conditions ranged from 0.14 - 0.81% with a grand mean of 0.46%. The mean selenium concentrations between stress and non-stress were not significant.

### 3.13 Mean polyphenolic compounds

The results for means with respect to phenolic acids, flavonoids and dpph (antioxidant activity) concentration are presented in Table 3.

**Table 3:** Mean concentrations of polyphenolic compounds (mg/l) and dpph (%) for cowpea inbred lines under non-stress and stress conditions.

family	Dba		Caf		chl		gal		syr	
	NS	S	NS	S	NS	S	NS	S	NS	S
279	79.94	163.03	102.66	200.19	96.61	214.68	95.19	282.14	94.51	212.58
38	95.87	230.80	121.35	279.76	119.24	311.00	131.03	434.64	117.14	308.90
406	132.29	196.73	164.11	239.76	171.00	262.58	212.97	357.97	168.90	260.48
503	88.71	86.73	112.95	110.63	109.07	106.26	114.91	110.47	106.97	104.16
20	95.62	72.90	121.06	94.40	118.89	86.61	130.47	79.36	116.79	84.51
78	84.51	227.47	108.02	275.85	103.10	306.26	105.47	427.14	101.00	304.16
408	61.55	70.80	81.06	91.93	70.47	83.63	53.80	74.64	68.37	81.53
28	60.36	95.37	79.77	120.77	69.59	118.54	52.41	129.91	66.82	116.44
189	182.90	104.26	223.53	131.21	242.93	131.17	326.86	149.91	240.83	129.07
84	71.17	173.15	92.37	212.08	84.16	229.07	75.47	304.91	82.06	226.97
131	414.76	82.41	495.70	105.56	572.40	100.12	848.53	100.75	570.30	98.02
255	447.04	73.15	533.52	94.69	617.90	86.96	920.19	79.91	615.56	84.86
75	237.22	81.30	287.29	104.25	320.12	98.54	449.08	98.25	318.02	96.44
353	87.47	116.73	111.50	145.85	107.31	148.89	112.14	177.97	105.21	146.79
57	69.20	162.78	90.05	199.90	81.35	214.33	71.03	281.58	79.25	212.23
325	102.66	75.87	129.32	97.87	128.89	90.82	146.30	86.03	126.79	88.72
396	72.04	68.21	93.38	88.89	85.38	79.95	77.41	68.80	83.28	77.85
142	380.43	76.98	455.41	99.18	523.63	92.40	771.30	88.53	521.53	90.30
223	61.42	71.05	80.92	92.22	70.30	83.98	53.53	75.19	68.20	81.88
230	81.94	55.67	105.00	74.26	99.45	62.16	99.69	41.08	97.35	60.83
55	80.80	63.64	103.67	83.53	97.84	73.45	97.14	58.53	95.74	71.35
116	240.31	62.10	290.92	81.79	324.51	71.35	456.03	55.19	322.41	69.25
398	62.90	471.98	82.66	562.80	72.40	653.33	56.86	976.30	70.30	651.00
186	80.80	144.76	103.67	178.74	97.84	188.72	97.14	241.03	95.74	186.62
Mean	140.60	126.27	173.85	157.02	182.57	162.20	231.55	199.21	180.57	160.21
Std.err	23.90	18.57	28.06	21.82	33.97	26.41	53.78	41.82	33.97	**26.41**
CV%	82.12	71.03	78.44	67.67	90.08	79.11	100.00	100.00	91.10	80.41
Min	60.36	55.67	79.77	74.26	69.59	62.16	52.41	41.08	66.82	60.83
Max	447.04	471.98	533.52	562.80	617.90	653.33	920.19	976.30	615.56	651.00
Sig	**ns**		**Ns**		**ns**		**ns**		**ns**	

family	Van		Pca		que		rut		dpph	
	NS	S	NS	S	NS	S	NS	S	NS	S
279	57.87	175.94	52.95	213.18	312.32	411.69	40.3	48.95	64.57	83.98
38	80.5	272.26	83.66	343.9	316.07	164.19	37.3	25.59	19.13	83.31
406	132.26	223.84	153.9	278.18	210.44	604.19	26.3	72.59	54.83	83.05
503	70.33	67.52	69.85	66.04	1764.5	70.38	153.45	13.14	79.03	81.84
20	80.15	47.87	83.18	39.3	216.69	176.07	29.59	26.16	0.57	78.94
78	64.36	267.52	61.76	337.47	359.19	431.07	47.09	48.37	39.4	76.63
408	31.73	44.89	17.47	35.33	293.57	227.32	33.95	32.16	32.78	74.25
28	30.19	79.8	15.62	82.71	182.94	222.32	17.71	31.09	81.25	72.58
189	204.19	92.43	251.52	99.85	1736.7	180.44	192.02	25.16	42.94	67.41
84	45.42	190.33	36.04	232.71	162.32	315.44	23.02	39.95	38.65	64.16
131	533.66	61.38	698.66	57.71	316.69	660.44	40.95	80.16	86.57	56.7
255	579.26	48.22	760.43	39.85	597.94	1629.8	74.66	151.87	53.61	55.67
75	281.38	59.8	356.28	55.56	1754.2	78.57	187.73	14.87	10.92	54.39
353	68.57	110.15	67.47	123.9	151.07	1649.8	19.59	143.3	60.76	53.14
57	42.61	175.59	32.23	212.71	199.82	1318.6	28.09	141.3	15.85	52.83
325	90.15	52.08	96.76	45.09	186.07	429.19	24.16	14.73	53.32	50.49
396	46.64	41.21	37.71	30.33	164.82	206.07	22.02	28.59	26.28	45.01
142	484.89	53.66	632.47	47.23	200.44	248.57	29.37	34.16	52.36	39.65
223	30.19	45.24	17.23	35.8	143.5	1963.9	19.09	172.02	44.21	23.19
230	60.71	23.53	56.8	6.57	218.57	1737.9	30.73	199.66	60.87	23.13
55	59.1	34.71	54.61	21.52	1089.8	1158.6	111.02	120.09	3.48	18.38
116	285.77	32.61	362.23	18.66	959.82	567.94	112.3	64.16	18.19	18.07
398	33.66	614.7	20.09	808.52	322.94	809.82	40.37	93.16	46.41	12.54
186	59.1	149.98	54.61	177.95	1761.7	1231.7	209.57	141.09	59.82	0.61
Mean	143.93	123.57	169.75	142.11	567.6	687.26	64.62	73.46	43.56	52.9
Std.err	46.1	35.84	33.97	26.41	120.72	122.19	12.5	11.68	4.89	**5.2**
CV%	100	99.9	100	103	103	86.77	93.12	77.14	54.34	47.17
Min	30.19	23.53	15.62	6.57	143.5	70.38	17.71	13.14	0.57	0.61
Max	579.26	614.7	760.43	808.52	1764.5	1963.9	209.57	199.66	86.57	83.98
Sig	**Ns**		**Ns**		**ns**		**ns**		**ns**	

dba = 2, 5-dihroxybenzoic acid, caf = caffeic acid, gal = gallic acid, chl = chlorogenic acid, pca = p-coumeric acid, syr = syringic acid, van = vanilic acid, que = quercetin, rut = rutin, NS = Non-stress condition, S=Stress condition, Std.err = Standard error (mean), Min = Minimum, Max = Maximum, CV = Coefficient of variation, ns = statistically not significant P > 0.05 using t-test Sig = significance level.

#### 3.13.1 Phenolic acids

Mean 2, 5-dihroxybenzoic acid concentration.

Mean 2, 5-dihroxybenzoic acid concentration for non-stress cowpea inbred lines ranged from 60.36 mg/l to 447.04 mg/l with a grand mean of 140.60 mg/l, while cowpea inbred lines under stress conditions recorded values which ranged from 55.67 mg/l to 471.98 mg/l with a grand mean of 126.27 mg/l. However, test of means for 2, 5-dihroxybenzoic acid concentrations for cowpea inbred lines under non-stress and stress conditions showed no significant differences.

#### 3.13.2 Mean caffeic acid concentration

The mean caffeic acid concentration for non-stress cowpea inbred lines ranged from 79.77 mg/l to 533.52 mg/l with a grand mean of 173.85 mg/l, whereas cowpea inbred lines under stress conditions ranged from 74.26 mg/l to 562.80 mg/l with a grand mean of 157.02 mg/l. Mean caffeic acid concentrations between stress and non-stress conditions were not significantly different.

#### 3.13.3 Mean gallic acid concentration

Mean gallic acid concentration for non-stress cowpea inbred lines ranged from 52.41 mg/l to 920.19 mg/l with a grand mean of 231.55 mg/l, whereas cowpea inbred lines under stress conditions ranged from 41.08-976.3 mg/l with a grand mean of 199.21 mg/l. The mean gallic acid concentrations between stress and non-stress were not significantly different.

#### 3.13.4 Mean chlorogenic acid concentration

Mean chlorogenic acid concentration for non-stress cowpea inbred lines recorded between 69.59-617.90 mg/l with a grand mean of 182.57 mg/l, whereas cowpea inbred lines under stress conditions ranged from 62.16-653.33 mg/l with a grand mean of 162.20 mg/l. The mean chlorogenic acid concentrations between stress and non-stress were not significantly different.

#### 3.13.5 Mean p-coumeric acid concentration

The minimum and maximum p-coumeric acid concentration was 15.62 mg/l and 760.43 mg/l respectively for cowpea inbred lines under non-stress conditions, with a grand mean of 169.75 mg/l. However, the mean p-coumeric acid for cowpea inbred lines under stress ranged from a value of 6.57 mg/l to 808.52 mg/l about a grand mean of 142.11 mg/l. The mean p-coumeric acid concentrations between stress and non-stress were not significantly different.

#### 3.13.6 Mean syringic acid concentration

Mean syringic acid concentration for non-stress cowpea inbred lines ranged from 66.82 mg/l to 615.56 mg/l with a grand mean of 180.57 mg/l, whereas cowpea inbred lines under stress conditions ranged from 60.83 mg/l to 651 mg/l with a grand mean of 160.21 mg/l. The mean syringic acid concentrations between stress and non-stress were not significantly different.

### 3.14 Mean vanilic acid concentration

Mean vanilic acid concentration for non-stress cowpea inbred lines ranged from 30.19 mg/l 579.26 mg/l with a grand mean of 143.93 mg/l, whereas cowpea inbred lines under stress conditions ranged from 23.53 mg/l to 614.70 mg/l with a grand mean of 123.57 mg/l. The mean vanilic acid concentrations between stress and non-stress were not significantly different.

### 3.15 Flavonoids

#### 3.15.1 Mean rutin concentration

Mean rutin concentration for non-stress cowpea inbred lines ranged from 17.71 mg/l to 209.57 mg/l with a grand mean of 64.62 mg/l, whereas that of cowpea inbred lines under stress conditions ranged from 13.14 mg/l to 199.66 mg/l with a grand mean of 64.62 mg/l. The mean rutin concentrations between stress and non-stress were not significantly different.

#### 3.15.2 Mean quercetin concentration

The minimum and maximum quercetin concentration was 143.50 mg/l and 1764.50 mg/l respectively for cowpea inbred lines under non-stress conditions, with a grand mean of 567.6 mg/l. However, the mean quercetin for cowpea inbred lines under stress ranged from a value of 70.38 mg/l to 1963.9 bmg/l with a grand mean of 687.26 mg/l. The mean quercetin concentrations between stress and non-stress were not significantly different.

### 3.16 Antioxidant activity

#### 3.16.1 Mean dpph concentration

The mean dpph concentration for cowpea inbred lines under stress ranged from a value of 0.57–86.57%. The minimum and maximum mean dpph was 0.61% and 83.98% respectively for cowpea inbred lines under non-stress condition. Correspondingly, cowpea inbred lines under stress scored a mean value of 52.9% higher than non-stress treatments, which scored 43.56% of dpph concentration.

### 3.17 Pairwise correlation coefficient between crude protein and mineral elements in cowpea inbred lines under non-stress and stress conditions

Pairwise correlation coefficient between crude protein and mineral elements among cowpea inbred lines are presented in Table 4. There were 10 and 12 significant associations between mineral elements and crude protein among cowpea inbred lines evaluated for both non-stress conditions and stress conditions respectively.

**Table 4:** Pairwise correlations among crude protein and mineral elements in cowpea inbred lines under non stress (below diagonal) and stress (above diagonal) conditions.

	P	CP	Mg	K	Cr	Zn	Fe	Pb	Cu	Mn	Se
P	-	-0.019	0.1122	0.2896*	0.1195	0.0124	0.0201	0.0035	-0.105	-0.2653*	0.061
CP	0.2405*	-	0.1056	0.3590*	0.0482	-0.098	-0.2329*	-0.019	-0.3652*	0.1324	0.1429
Mg	0.0453	0.0293	-	0.7091*	-0.006	0.6845*	0.1659	-0.071	0.0306	0.037	0.0672
K	-0.065	-0.027	0.6298*	-	-0.144	0.2737*	-0.007	-0.024	-0.069	0.0114	0.0355
Cr	-0.047	-0.134	0.1298	0.2953*	-	-0.089	0.1036	-0.196	-0.088	0.1031	-0.052
Zn	0.3349*	-0.14	0.5650*	0.3189*	0.1351	-	0.3958*	-0.187	0.0882	-0.182	0.0225
Fe	0.0351	0.2590*	0.1562	0.1727	0.1476	0.2869*	-	-0.4711*	0.158	-0.133	0.3806*
Pb	0.0505	-0.142	0.0907	0.0427	0.1687	-0.159	-0.064	-	-0.036	0.175	-0.156
Cu	0.2546*	-0.118	0.1004	0.1584	-0.163	0.1964	0.1811	0.0093	-	-0.149	-0.2455*
Mn	.	.	.	.	.	.	.	.		-	0.0653
Se	0.2085	-0.201	-0.101	-0.054	-0.157	0.0899	-0.3504*	-0.034	0.2279	-	-

P=Phosphorus, CP = Crude protein, Mg = Magnesium, K=Potassium, Cr=Chromium, Zn = Zinc, Fe=Iron, Pb = Lead, Cu=Copper, Mn = Manganese, Se= Selenium, * = significant at 0.05.

For non-stress, phosphorus was significantly associated with crude protein and copper comparatively, under stress conditions phosphorus was significantly associated with potassium and manganese. Crude protein significantly correlated with iron only under non-stress conditions. Contrastingly, under stress conditions crude protein significantly associated with Cu. For both stress and non-stress conditions, Mg positively correlated with K and Zn under stress conditions. Potassium was significant and positively correlated with Cr and under non-stress conditions. On the other hand, under stress conditions, potassium was only significant and positively correlated with Zn. Under non-stress condition, zinc was significant and positively associated with only Fe whereas significant and positively correlated with Fe under stress condition. There was significant association between Fe and Se under non-stress conditions. Also, Fe was significant and associated with Pb and Se under stress conditions. Under stress conditions, Cu was significant and negatively associated with Se.

### 3.18 Pairwise correlations among polyphenolic compounds and dpph in cowpea inbred lines under non-stress and stress conditions

Pairwise correlation coefficient between polyphenolic compounds and dpph among are presented in Table 5. Correlation between polyphenolic compounds and dpph among cowpea inbred lines evaluated under non-stress and stress showed 23 and 24 significant associations respectively. At significance of P ˂ 0.001, there were perfect correlations among the 7 phenolic acids for both non-stress and stress conditions. The flavonoids; quercetin and rutin were highly significant and positively associated with each other under non-stress and stress conditions. Under stress conditions, quercetin and rutin were significantly and negatively correlated with dpph (see Tables 5, 6 and 7).

**Table 5:** Pairwise correlations among polyphenolic compounds and dpph in cowpea inbred lines under non-stress (below diagonal) and stress (above diagonal) conditions.

	dba	caf	chl	gal	syr	Pca	van	que	rut	dpph
Dba	**-**	1.0000*	1.0000*	1.0000*	1.0000*	1.0000*	1.0000*	-0.056	-0.013	-0.023
Caf	1.0000*	-	1.0000*	1.0000*	1.0000*	1.0000*	1.0000*	-0.056	-0.013	-0.023
Chl	1.0000*	1.0000*	-	1.0000*	1.0000*	1.0000*	1.0000*	-0.056	-0.013	-0.023
Gal	1.0000*	1.0000*	1.0000*	-	1.0000*	1.0000*	1.0000*	-0.056	-0.013	-0.023
Syr	1.0000*	1.0000*	1.0000*	1.0000*	-	1.0000*	1.0000*	-0.056	-0.013	-0.023
Pca	1.0000*	1.0000*	1.0000*	1.0000*	1.0000*	-	1.0000*	-0.056	-0.013	-0.023
Van	1.0000*	1.0000*	1.0000*	1.0000*	1.0000*	1.0000*	-	-0.056	-0.013	-0.023
Que	0.1206	0.1206	0.1206	0.1206	0.1206	0.1206	0.1206	-	0.9764*	-0.5636*
Rut	0.1555	0.1555	0.1555	0.1554	0.1555	0.1555	0.1555	0.9886*	-	-0.5888*
Dpph	0.1773	0.1773	0.1773	0.1772	0.1773	0.1773	0.1773	-0.06	-0.086	-

* = significant at 5% level, dba = 2, 5-dihroxybenzoic acid, caf = caffeic acid, gal = gallic acid, chl = chlorogenic acid, pca = p-coumeric acid, syr = syringic acid, van = vanilic acid, que = quercetin, rut = rutin.

**Table 6:** Significant associations between yield component, crude protein, mineral elements, polyphenolic compounds and yield components among cowpea inbred lines under non-stress condition.

	CP	Mg	K	Zn	Dpph
glu	0.6093**				
dpl	0.5397**			-0.4421*	
lva	0.5538**				
lty	0.6014**				
thp	0.4215*		-0.4584*		
ltp	0.5067**				
que		0.4315*			
rut		0.4402*			
lly		-0.4521*	-0.4799*		
lmh		-0.4762*			
bth			-0.4150*		
lth			-0.4310*		
lhs					-0.5393**
lse					-0.6095**

* = significant at 5%, ** = significant at 1%, P=Phosphorus, CP = Crude protein, Mg = Magnesium, K=Potassium, Zn = Zinc, glu = Glutamine, lly = L-lysine, lhs = L-histidine, lse = L-Serine, bth = B-Threonine, lth = L-Threonine, dpl = D-Proline, lva = L-Valine, lmh = L-Methionine, lty = L-Tyrosine, thp = Trans-4-Hydroxy-L-Proline, ltp = L-Tryptophan.

**Table 7:** Significant associations between yield component, crude protein, mineral elements, polyphenolic compounds and yield components among cowpea inbred lines under stress conditions.

	P	CP	K	Cr	Fe	Cu
lse	0.4488*					
gly	0.4299*	0.4514*				
glu		0.5436**	0.4941**			
lly				0.5057**		0.5099**
lmh					0.4699*	
dla						-0.5581*

* = significant at 5% level, ** = significant at 1% level, P=Phosphorus, CP = Crude protein, Mg = Magnesium K=Potassium, Cr=Chromium, Fe=Iron, Pb = Lead, Cu=Copper, glu = Glutamine, lly = L-lysine, dla = DL-Alpha-Alanine, lse = L-Serine, gly = Glycine, lmh = L-Methionine.

### 3.19 Principal component analysis of crude protein and mineral elements

The Principal component analysis of crude protein and mineral elements are presented in Table 8. Five principal components were identified in both non-stress and stress conditions and accounted for 81.51% and 80.08% correspondingly of the total variation. The first PC for non-stress had an eigenvalue of 2.44 accounted for 27.12 % of the total variation whereas stress conditions had an eigenvalue of 2.48 which accounted for 24.3% of total variation. The second and third PC axes with eigenvalues of 1.67 and 1.47 accounted for 18.57% and 16.36% respectively of the total variation for non-stress conditions. The second and third PC axes with eigenvalues of 2.12 and 1.56 accounted for 21.18% and 15.61% respectively of the total variation for stress conditions. However, components 4 and 5 accounted for 10.64% and 8.82% of total variation for non-stress conditions, whereas for stress conditions both components scored 10.62% and 7.84% respectively of total variation for the 4^th^ and 5^th^ PC's.

**Table 8:** Principal component analysis of crude protein and mineral elements.

Variable	Eigenvectors									
	Non-stress					Stress				
	PC1	PC2	PC3	PC4	PC5	PC1	PC2	PC3	PC4	PC5
CP	-0.02	**-**0.42	0.44	0.09	0.61	0.20	0.45	-0.06	-0.28	-0.24
Mg	0.55	-0.08	-0.11	0.13	0.27	0.51	0.25	-0.16	0.10	0.34
K	0.51	0.04	-0.12	**-**0.30	0.36	0.44	0.35	-0.32	-0.03	0.08
Cr	0.41	-0.11	**-**0.32	-0.33	-0.17	-0.04	0.22	0.48	-0.47	0.55
Zn	0.27	0.48	0.30	-0.16	-0.22	0.43	-0.32	-0.21	0.12	-0.06
Fe	0.34	-0.10	0.51	0.24	-0.27	0.38	-0.36	0.34	0.01	0.06
Pb	0.11	-0.13	-0.52	0.65	-0.05	-0.31	0.13	-0.37	0.41	0.31
Cu	0.17	0.52	0.16	0.51	0.12	-0.02	-0.43	-0.29	-0.14	0.59
Mn	**.**	.	.	.	.	-0.09	0.36	0.26	0.52	0.24
Se	-0.21	0.52	-0.19	-0.09	0.51	0.26	-0.08	0.45	0.46	-0.02
Eigenvalue	2.44	1.67	1.47	0.96	0.79	2.48	2.12	1.56	1.06	0.78
Difference	0.77	0.20	0.52	0.16	0.21	0.36	0.56	0.50	0.28	0.14
Proportion	27.12	18.57	16.36	10.64	8.82	24.83	21.18	15.61	10.62	7.84
Cumulative	27.12	45.69	62.05	72.69	81.51	24.83	46.01	61.62	72.24	80.08

P=Phosphorus, CP=Crude protein, Mg=Magnesium, K=Potassium, Cr=Chromium, Zn=Zinc, Fe=Iron, Pb=Lead, Cu=Copper, Mn=Manganese, Se= Selenium.

The first principal component with reference to its high factor loadings was positively associated with magnesium, potassium, iron and copper contributing, for both stress and non-stress conditions. Additionally, under stress conditions, the 1^st^ PC were also accounted for by zinc and lead. The second principal component under both non-stress and stress conditions were associated with crude protein, zinc and copper Furthermore, the second PC under stress conditions were also contributed by potassium, iron, copper and manganese.

Most of the factor loadings in the third principal components of both conditions were mostly accounted for by potassium, chromium, iron and lead. The 4^th^ principal component for non-stress conditions were positively accounted by lead and copper. For stress conditions, the 4^th^ PC was highly accounted for by manganese, selenium, lead and chromium. Finally, the 5^th^ principal component for non-stress were mostly attributed to crude protein, potassium and selenium, whereas under stress conditions magnesium, chromium, lead and copper dominated variation in that component.

### 3.20 Principal component analysis of polyphenolic compounds under non-stress and stress conditions

The Principal component analysis of polyphenolic compound concentration are presented in Table 9. There was a total of four principal components identified for both non-stress and stress conditions account for 100% of the total variation. The first PC for non-stress had an eigenvalue of 7.09 accounted for 70.87% of the total variation whereas that for stress conditions scored an eigenvalue of 7.01 which accounted for 70.08% of total variation. The second and third PC axes with eigenvalues of 1.01 and 0.93 accounted for 19.58% and 9.44% respectively of the total variation for non-stress conditions. The second and third PC axes with eigenvalues of 1.96 and 0.48 accounted for 24.65% and 5.05% respectively of the total variation for stress conditions. Finally, the 4^th^ PC had an eigenvalue of 0.01 and 0.02 accounted for 0.10% and 0.22% respectively of the total variation for non-stress and stress conditions.

**Table 9:** Principal component analysis of polyphenolic compounds concentration evaluated under non-stress and stress conditions.

Variable	Eigenvectors							
	Non-stress				Stress			
	PC1	PC2	PC3	PC4	PC1	PC2	PC3	PC4
dba	0.38	-0.03	-0.03	0.00	0.38	0.00	0.01	0.00
caf	0.38	-0.03	-0.03	0.00	0.38	0.00	0.01	0.00
chl	0.38	-0.03	-0.03	0.00	0.38	0.00	0.01	0.00
gal	0.38	-0.03	-0.03	0.00	0.38	0.00	0.01	0.00
syr	0.38	-0.03	-0.03	0.00	0.38	0.00	0.01	0.00
pca	0.38	-0.03	-0.03	0.00	0.38	0.00	0.01	0.00
van	0.38	-0.03	-0.03	0.00	0.38	0.00	0.01	0.00
que	0.06	0.70	0.12	0.70	-0.02	0.61	0.38	-0.70
rut	0.08	0.70	0.08	-0.71	-0.01	0.62	0.33	0.72
dpph	0.07	-0.15	0.99	-0.02	-0.03	-0.50	0.86	0.04
Eigenvalue	7.09	1.96	0.94	0.01	7.01	2.46	0.51	0.02
Difference	5.13	1.01	0.93	0.01	4.54	1.96	0.48	0.02
Proportion	70.87	19.58	9.44	0.10	70.08	24.65	5.05	0.22
Cumulative	70.87	90.46	99.90	100.00	70.08	94.73	99.78	100.00

dba = 2, 5-dihroxybenzoic acid, caf = caffeic acid, gal = gallic acid, chl = chlorogenic acid, pca = p-coumeric acid, syr = syringic acid, van = vanilic acid, que = quercetin, rut = rutin.

The first principal component with reference to its high factor loadings was positively and highly associated with the 7 phenolic acids (2, 5-dihroxybenzoic acid, caffeic acid, chlorogenic acid, gallic acid, p-coumeric acid, syringic acid and vanilic acid) for both stress and non-stress conditions. The second PC for both non-stress and stress conditions were highly associated with the flavonoids (quercetin and rutin) and additionally, dpph for stress conditions. The third PC for both stress and non-stress condition was mainly and positively accounted for by dpph. The 4th PC for both stress and non-stress showed that the flavonoids accounted for the variations.

## 4 Discussion

In the present study, the mean crude protein content under non-stress and stress condition were 20.69 and 20.34%. The level of variation within the inbred lines ranged from 14.58-47.90% and 11.60–30.23% under non-stress and stress respectively. In a study of mean crude protein in cowpea, Evans and Boulter (1974) recorded mean crude protein content that ranged from 22% and 24%, but varied from 21% up to 34% in other crop species which is in agreement with this present study.

Results in this current study revealed that water-stress did not have any significant effect on the concentration of the following mineral elements; calcium (Ca), copper (Cu), iron (Fe), potassium (K), magnesium (Mg), manganese (Mn), sodium (Na), phosphorus (P), zinc (Zn), Chromium (Cr), and Selenium (Se). However, (Gerrano et al., 2018) in a similar study reported that differential irrigation treatments significantly affected concentrations of Nitrogen, Calcium, Magnesium, Copper, Bromine, and Iron in the field grown cowpea seeds and under field conditions. These differences may be due to genotypic differences in cowpea, environmental condition, and or laboratory procedures/protocols used for the study. In this present study, magnesium showed very strong significant correlation with potassium and zinc for both non-stress and stress conditions. This corroborates drought study in potato by (Lefèvre et al., 2012), who reported that Mineral concentrations in potato tubers were highly variable among genotypes, they concluded that some were significantly and positively correlated with each other, the most remarkable associations were between Na and Ca, Mn and Mg and Zn and Fe, in both control and drought-stressed plants.

In this current study, there was perfect correlation among the phenolic acids and very strong associations among the flavonoids under both treatments. It must be added that, there was fairly strong association between flavonoids and antioxidants under stress conditions. This may be due to the fact that, some of the flavonoids; quercetin and rutin were more abundant in stress inbred lines than non-stress inbred lines.

## 5 Conclusion

This study showed that seeds of cowpea are very good source of protein and mineral elements, polyphenolic compounds, and antioxidants. The significant differences in water regimes (stress and non-stress) on various phytochemical traits were only observed in phosphorus, and lead. It must be highlighted that cowpea inbred, family number 57 subjected to non-stress treatment had a very high amount of crude protein content of 46.90%. Inbred line with family number 84 under water stress had the maximum concentration of Mg and K. Family 20 under well-watered condition had the maximum antioxidant content. For phenolic acid content, family 255 scored the maximum. Quercetin and rutin were most abundant in families IT93K-503-1 and 186 respectively under both contrasting conditions. Hence, the need to consider these varieties for selection for cowpea breeding programmes on nutritional basis. A perfect correlation existed among the phenolic acids. Strong significant correlation between the flavonoids indicate that each compound within the separate polyphenolic compound component is dependent on the other. Various groups of phytochemical traits may contribute to genetic variation. Furthermore, some phytochemical traits may be strongly linked to each other. Cowpea can be a great source of polyphenolic compounds and antioxidants as compared to other whole grains, fruits and vegetables. The study therefore concludes that water stress has no significant effects on the nutritional components of the developed cowpea recombinant line used in the study.

## Declarations

### Author contribution statement

Alidu, M.S.: Conceived and designed the experiments; Performed the experiments; Analyzed and interpreted the data; Wrote the paper.

Asante, I.K., Mensah, H.K.: Contributed reagents, materials, analysis tools or data.

### Funding statement

This work was supported by the Alliance for a Green Revolution in Africa (AGRA), Ghana via a scholarship for my PhD study at the West Africa Centre for Crop Improvement (WACCI), University of Ghana, Legon.

### Competing interest statement

The authors declare no conflict of interest

### Additional information

Data associated with this study has been deposited at Dryad Data repository under the https://doi.org/10.5061/dryad.45q3c56.
